# Harnessing Ultrasonic Technologies to Treat *Staphylococcus Aureus* Skin Infections

**DOI:** 10.3390/molecules30030512

**Published:** 2025-01-23

**Authors:** John Hulme

**Affiliations:** Department of Bionano Technology, Gachon Bionano Research Institute, Gachon University, 1342 Sungnam-daero, Sujung-gu, Seongnam-si 461-701, Republic of Korea; flp15@gachon.ac.kr

**Keywords:** ultrasound, microbubble, sonoporation, skin infections, antibiotic resistance

## Abstract

The rise of antibiotic-resistant *Staphylococcus aureus* strains, particularly MRSA, complicates the management of skin and soft tissue infections. This review highlights ultrasonic methodologies as adjunctive therapies to combat *S. aureus*-driven skin infections and prevent progression to biofilm formation and chronic wounds. Low- and high-frequency ultrasound (LFU and HFU) demonstrate potential in disrupting biofilms, enhancing drug delivery, and promoting tissue repair through cavitation and microbubble activity. These approaches integrate ultrasonic frequencies with microbubbles and therapeutics, such as antibiotics and affimers, to minimize resistance and improve healing. Tailoring the bioeffects of ultrasound on skin structures through localized delivery technologies, including microneedle patches and piezoelectric systems, presents promising solutions for early intervention in skin and soft structure infections (SSSIs).

## 1. Introduction

A significant majority (approximately 90%) of *Staphylococcus aureus* infections are skin and soft structure infections (SSSIs), making up the primary portion of staphylococcal disease [1]. Due to *S. aureus*’s ability to form biofilms, which enhances its antimicrobial resistance and protects it from host immune responses, treatments remain challenging. Methicillin-resistant *S. aureus* (MRSA) exacerbates these challenges, with resistance mechanisms such as the production of penicillin-binding protein 2a (PBP2a), encoded by the mecA gene within the staphylococcal chromosome cassette mec (SCCmec) [2]. The global rise of antibiotic-resistant strains, including MRSA, vancomycin-intermediate S. aureus (VISA), and vancomycin-resistant *S. aureus* (VRSA), has necessitated the prioritization of novel therapeutic strategies.

Compounding these challenges is the composition and metabolic heterogeneity of biofilms, which act as protective barriers, reducing antibiotic effectiveness by up to 1000-fold compared to planktonic bacteria [3]. Current treatment approaches, such as invasive sharp debridement, aim to remove biofilms and promote wound healing. However, the presence of (metabolically dormant) persister cells within biofilms often leads to biofilm regeneration, undermining the efficacy of debridement and delaying wound closure. Thus, non-invasive therapies are urgently needed to address these limitations.

Low-frequency ultrasound (LFU) has emerged as a promising non-invasive therapy for biofilm-associated chronic wounds. By leveraging cavitation and acoustic energy, LFU disrupts biofilms, enhances antibiotic penetration, and promotes tissue repair. Commercial devices, such as Soering and Arobella, have demonstrated similar efficacy to standard wound debridement in clinical trials, highlighting their potential as a non-invasive, patient-friendly alternative to conventional physical treatments for *S. aureus* skin infections [4,5,6].

This review focuses on the application of ultrasonic methodologies to address key challenges in *Staphylococcus aureus*-driven skin infections, with an emphasis on preventing skin dysbiosis, biofilm formation, and intracellular infection. By exploring mechanisms such as cavitation, sonoporation, and microbubble technologies, this review evaluates their potential as adjunctive therapies for enhancing treatment efficacy, reducing antibiotic resistance, and promoting rapid infection resolution [7]. Finally, integrated non-invasive delivery methods are discussed, including microneedle patches and piezoelectric systems, which hold promise in minimizing off-target complications associated with SA skin infections [4,8].

## 2. Cavitation

Ultrasonic cavitation, a key mechanism underpinning advanced delivery methods, leverages ultrasound-driven microbubble activity to disrupt biofilm structure and improve therapeutic delivery. Stimuli-responsive drug delivery systems, such as low- and high-frequency ultrasound (LFU and HFU), harness this process to improve the efficacy of conventional antibiotics and limit resistance [9,10,11]. These systems also extend their benefits to biological therapies, including antibodies and CRISPR-based treatments, offering versatile strategies to address multidrug-resistant *Staphylococcus aureus* infections [12,13]. The ability of either LFU or HFU to enhance therapeutic efficacy rests in a pressure-induced phenomenon known as cavitation, which can induce various bubble characteristics such as nucleation, excitation, oscillation, and collapse in fluids [14]. Bubble collapse is often associated with unstable (inertial) cavitations resulting from higher US pressures and intensities, whereas repetitive oscillatory symmetric behaviors are seen with stable (non-inertial) cavitations at low pressures.

**Figure 1:**
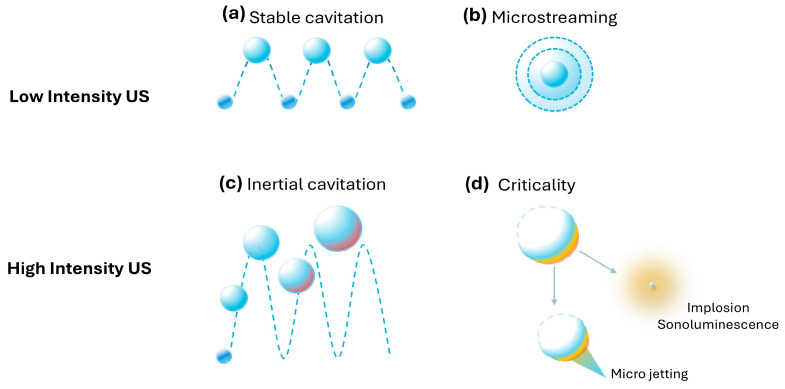
Principles and mechanisms of continuous low and high-intensity ultrasound. (**a**) Stable cavitation refers to the oscillation of gas bubbles in a liquid of known viscosity under the influence of low-intensity ultrasound (US). The bubbles expand and contract rhythmically without collapsing, creating micro-level mechanical effects. (**b**) Microstreaming describes the small-scale fluid movement caused by the oscillations of stable cavitation bubbles. This phenomenon enhances mixing and can influence cellular and molecular processes. (**c**) Inertial cavitation occurs when bubbles generated by high-intensity US collapse violently after expanding to a critical size. This results in localized shock waves and high-energy release. (**d**) Criticality represents the extreme outcomes of inertial cavitation, such as implosion, which generates high temperatures and pressures, sonoluminescence, the emission of light during bubble collapse, and microjetting, where focused liquid jets are ejected, causing significant localized impact.

It has been postulated that microbubbles in stable cavitation may cause the surrounding fluid to adopt diverging flow patterns or microstreams (acoustic streaming) [15]. In addition, these microstreams can encourage circular flow, enhancing shear stresses for nearby cells and changing their membrane packing density and porosity, a process termed “sonoporation” [16,17]. Moreover, regulated streaming [18,19] can influence microbubble shedding events, aiding smaller vesicle distribution, which can be beneficial by increasing the available surface area of therapeutic agents and enhancing cellular uptake. On the other hand, bubble collapse or implosion can occur either symmetrically or asymmetrically, releasing the excess energy in the form of heat [20], light [21], shockwave [22], microjets [23], and reactive oxygen species [24] (Figure 1). Collectively, when combined with supplementary (exogenous) MBs, ultrasound-induced cavitations are considered one of the most versatile tools available in targeted drug delivery.

US cavitations can also be induced with US alone via the direct nucleation of gases dissolved in bodily fluids [25]. This unpredictable endogenous process requires high-intensity ultrasound (HIU), which, when unfocused [26], can result in non-localized tissue damage. The risk of tissue damage can be mitigated by priming the target with nucleation sites, allowing for lower intensities. A similar approach can also be used to modulate the uptake and penetration of hydrophilic and hydrophobic antibiotics across bacterial membranes [27]. Antibiotics, in combination with US and cavitation nuclei, fall under the nomenclature of sono-bactericides. In addition to sono-bactericides, the modulating actions of cavitating nuclei have found usage in the areas of thrombolysis (sono-thrombolysis) [28], reperfusion (sono-reperfusion) [29], and cancer therapy (sonodynamic therapy) [30,31,32]. The broad application of cavitating nuclei (CN) rests in the properties of the numerous micro-nano bubble compositions available.

### 2.1. Micro-Nano Bubble Compositions

Initially developed in the 1970s and 1980s [33] for medical imaging applications, gas-filled microbubbles represent some of the oldest and most versatile CN still in use. The imaging versatility provided by these nuclei lies in a combination of a highly compressible hydrophobic gas (sulphur hexafluoride, perfluorocarbon, or octafluoropropane) encapsulated by a lipid-rich stabilizing shell. This has prompted a range of gas microbubble sizes (1–10 μm) to be investigated and their performance characteristics and potential for commercialization to be assessed [34].

After numerous successful large-scale safety trials involving three different gas–shell microbubble (GSM) compositions, the generic names of SonoVue, Definity, and Sonoazoid have established themselves within the field of ultrasonic medical imaging as reliable contrast agents [35]. A component of that success has been the inclusion of various stabilizing phospholipids, such as distearoylphosphatidylcholine (DSPC) and dipalmitoyl phosphatidylcholine (DPPC), in MB formulations [36]. These lipids’ molecular (~3 nm monolayer) organization and the varying stabilities afforded to the gaseous phase by the differing acyl chain length are well-established [37]. In short, the longer the acyl chain lengths, the greater the packing density, the lower the gas permeability (leakage), and the longer the storage lifetime [38]. The clinical success of these agents lies in their ability to produce a high-resolution image in a short time frame (seconds to minutes), limiting physiological and adverse side effects (ASE) [39].

In addition to phospholipids, protein shells composed of human serum albumin (HSA) have led to the FDA approval of GSM contrast agents Albunex and Optison [40,41,42]. In research settings, the cheaper alternative to HSA, bovine serum albumin (BSA), is used to elucidate the roles of disulfide bonding in the forming and degrading MB protein shells. Investigations by Bussonniere et al. [40] demonstrated the importance of available thiol groups and their impact on crosslinking density and compliance. The potential costs of enhanced compliance are increased resistance to dissolution, cavitation inhibition, and susceptibility to acoustic dampening [42].

Depending on the physical requirements, microbubbles are compatible with various polymer shells [43]. In particular, the versatility of poly (butyl cyanoacrylate) (PBCA) has been utilized to produce shell rigidities exceeding that of lipid and protein bubbles. However, the high acoustic dampening accompanying shell rigidity can result in a high-velocity gas ejection when the bubble is exposed to high ultrasonic pressures [44]. At present, no polymer MBs have been clinically approved. On the other hand, PBCA has found usage as a model polymer in poly (vinyl alcohol) (PVA) film-stretching technologies, permitting the enhanced permeability and long circulatory lifetimes of anisotropic (oval) bubbles to be studied [45] in murine models.

Evolutionary additions to conventional microbubble formulations are nanodroplets (NDs), nanobubbles (NBs), and bacterial vesicles (BVs). NDs’ fabrication, storage, and applications are well-reported [46,47,48]. Traditionally, NDs consist of a metastable superheated liquid (perfluoro pentane) core enclosed by a stabilizing polymer, lipid, or protein shell [49,50,51,52]. Upon exposure to ultrasonic pressure, the liquid vaporizes, driving droplet expansion via a process known as acoustic droplet vaporization (ADV). Investigations of nano-microbubble (416 nm–8 μm) expansions report microbubbles showing similar cavitational and echogenic properties to commercially available contrast agents under physiological conditions [53]. The clinical potential of nano-micro droplet phase transitions has been demonstrated in the diagnosis of skin melanomas and other tumors [54,55]. As a side note, the recent work by Vasiukhina et al. [53] suggested that echogenic enhancement may be achievable without ADV. Using 300 nm tridecafluorohexane (TDFH) cores and BSA shells, the group demonstrated that the echogenic properties of 13-h-old nanodroplets could be enhanced by an order of magnitude in the absence of ADV with minimal expansion. The authors suggested that further work should include in vivo models, ensuring theoretical predictions concur with experimental outcomes.

**Figure 2:**
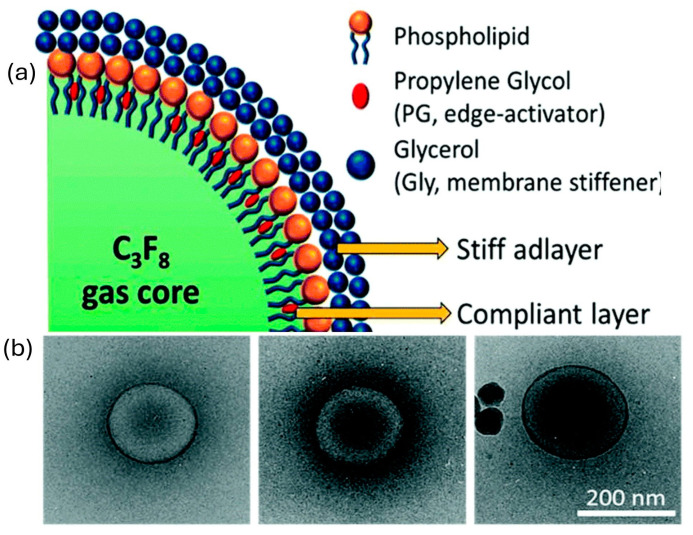
A schematic of the ultrastable NBs. (**a**) A schematic depiction of the ultrastable nanobubble (NB) shell, illustrating its bilayer architecture with regions of elastic contrast. The shell also contains DSPE-mPEG 2000, which is not represented in the schematic. (**b**) Cryo-electron microscopy (cryo-EM) images of PG-Gly-PL, highlighting the nanobubble membrane and its dense C3F8 gas core. Reproduced [58] with permission from the Royal Society of Chemistry.

Consisting of an echo-contrast gas and various mono and bilayer configurations, nanobubbles have demonstrated multifunctional capabilities acting as drug delivery vehicles and stable imaging contrast agents in vitro [56,57]. However, when subjected to the continuous insonation and extreme oscillations encountered in vivo, NBs exhibit reduced lifetimes and poor signal performance. A potential solution to these limitations was proposed by Leon et al. [58], in which the contrasting elastic properties common to multilayer bacteria envelopes and human skin were incorporated into the construction of a biomimetic (propylene glycol-glycerol-phospholipid) PG-Gly-PL shell (Figure 2). In theory, an artificial shell with biomimetic features would result in the NBs dissipating excess energy and stress in a similar manner to their natural counterparts, thereby extending their half-life. Upon examination, the resultant biomimetic perfluoropropane NBs exhibited ultra-stable resilience and toughness with an in vivo half-life of 18.32 ± 0.40 min. A schematic of the ultrastable NBs is depicted in Figure 2.

In addition to nanobubbles, the composition of cylindrical bacterial gas vesicles (GVs) also demonstrates enhanced stability [59,60]. With diameters ranging from 45–250 nm and typical lengths of 100–600 nm, said vesicles allow the free movement of gas from the interior to the exterior across a 2 nm thick protein shell. The resulting configuration negates potential pressure gradients and nanovesicle instability, prolonging half-life [61]. Different GV compositions are currently being explored in medical imaging, disease treatments, and drug delivery research applications [62].

Other vesicles of note are echogenic bi-layer liposomes (acoustically active liposomes) and invasomes that demonstrate enhanced drug-loading capacity [63] and increased permeability, respectively [64,65]. Similar in scale (30–400 nm), invasomes are liposomes consisting of unsaturated phospholipids, water, and ethanol-terpene/ol solvents that synergistically impart fluidity to the SC, improving drug partitioning into the skin. Early work by Tawfik et al. assessed the active skin permeation (sonophoresis) of 16 invasomes ranging in particle size (PS) from 131.9–867.0 nm loaded with the antidepressant drug Agomelatine (AGM) in rabbits. With an entrapment efficiency of 78.6%, PS of 313 nm, and an enhancement ratio of 4.83, combined with a duty cycle of 100% for 15 min in continuous mode, the authors demonstrated the limonene (1.5%)-based invasome “I2-LFU-C4” as a promising transdermal system for AGM [65]. In the absence of US, an invasome study showed their application could improve the treatment of Staphylococcus aureus infections. In 2024, researchers developed erythromycin stearate-loaded invasomes using soy lecithin combined with terpenes such as citral and eugenol. The invasomes demonstrated significant antibacterial activity against S. aureus, with zones of inhibition ranging from 15 to 20 mm [66]. The study concluded that invasomes could effectively enhance drug penetration to treat deep skin infections caused by S. aureus. In addition to skin permeability and antimicrobial [67] properties, terpene-invasomes strongly afford anti-inflammatory and oxidant properties to human skin epidermal keratinocytes (HaCaT cells). However, toxicity and stability questions remain, limiting further investigations with low-frequency ultrasound (sonophoresis) [67]. For those readers interested in the acoustic behavior of solid vesicles such as gold and mesoporous silica nanoparticles, the reviews by Manzano et al. and Goughari et al. are recommended [68,69]. These materials exhibit unique properties that differentiate them from traditional cavitating nuclei, broadening their potential applications in ultrasonic technologies.

### 2.2. Imparting Biocompatibility to Lipid and Protein Shells

Biocompatibility is critical to the lifetime of lipid and protein shell-based cavitating nuclei. Several surface modification strategies can be utilized to render lipid shells biocompatible with their host and less prone to reticuloendothelial entrapment and first-pass metabolism. The strategy most frequently employed involves the surfactant polyethene glycol (PEG), which can sterically shield lipid antigens from phagocytic cells and provide a conjugation bridge to liposomes, targeting ligands, antibodies, and others [70]. Lipids can also be modified with functional groups such as biotin, thiols, and amines prior to MB synthesis [71], permitting the inclusion of additional conjugation steps. However, in some individuals with repeated exposure to PEG-MB injections, anti-polyethylene glycol (PEG) may present with the potential for an anaphylactic reaction. In such circumstances, the cellular membrane components of the host can be incorporated into the initial formulations to improve immuno tolerance [72]. Additionally, electrostatic attraction is another method, where drugs and anionic nucleic acids directly attach to charged lipid shells, although questions persist over their reliability in circulation [73].

Protein shells contain a range of amino acids, including lysine (amine groups), aspartic (carboxylic acids), and cysteine (thiol), which can serve as natural conjugation sites [74] and, depending on the source, can exhibit varying degrees of immuno-compatibility. As of 2015, in 94 published papers, 41.5% employed maleimide thiol and specific lipid–peptide linkage strategies [75,76], with the remaining utilizing streptavidin/avidin-biotin covalent bridges [77]. In addition, protein shells composed of HSA can encapsulate various synthetic therapeutic peptides and proteins prior to MB formation, further improving compatibility [78]. The benefits of these approaches are best summarized by Lee et al. [52], in which HSA–PEG conjugates were shown to stabilize the properties of MB and nanodroplet shells, permitting their potential usage in murine models over 10 days.

## 3. Bioeffects on Skin

In the last thirty years, the bioeffects of ultrasound and its potential as a non-invasive drug delivery vehicle/adjuvant in skin and wound therapeutics have garnered significant interest [79,80]. The biological effects of ultrasound result from the attenuation of mechanical energy, mostly via adsorption, inducing particle oscillations in tissues with heat production. In addition, other factors such as convection, conduction, and ultrasonic parameters can influence biological temperature and skin permeability. The thermal effects of ultrasound are thought to play a positive role in sonophoretic (SP) drug–skin interactions, although doubts remain about the practical limitations of SP and its impact on drug diffusion. There are many obstacles to efficacious transdermal drug delivery, such as stratum corneum (SC) thicknesses (local), epidermal stratification, and keratinocyte differentiation, which are essential to skin integrity. Skin integrity is maintained via the continuous proliferation and migration of mature keratinocytes to the outer epidermal layer (SC). The resulting structure is a 15–20 μm thick brick-and-mortar configuration of keratin-filled dead corneocytes embedded in a liquid-filled matrix, excluding all but the smallest lipophilic molecules (<500 Da) [81].

According to the literature, the bioeffects frequently employed in skin permeability investigations are acoustic cavitation and microstreaming. Acoustic cavitation utilizes shockwaves and the microjets generated from symmetric or asymmetric bubble collapse, which temporarily perturb the SC, encouraging small channels to form and thereby increasing drug diffusion. Acoustic cavitation can be induced via a range of frequencies of low (20–100 kHz, coupling medium required) [82,83], intermediate (0.100–1 MHz), and high (>1 MHz). Each frequency range is reported to enhance skin permeability via different mechanisms. Another mechanism attributed to increased permeability is microstreaming, which generates shear stress and subsequent changes in the lipid density of the SC, providing easier passage for drugs to push through the skin.

When it comes to transdermal drug delivery, the choice of single or dual low frequency can play a significant role in drug permeability and skin integrity [84]. Some of the initial work involving single frequency (20 kHz) and various coupling mediums on drug skin permeability was performed by Pereira et al. [83]. The authors investigated the coupling performance of several media (synthetic surfactant poloxamer, hydroxyethyl cellulose, skin-enhancer chitosan, and carbopel hydrogels) on the number of localized transport regions (LTRs). Using the model drugs calcein and doxorubicin (DOX), the researchers observed an increase in LTR areas with LFU/Carbopol/ poloxamer treatments and an increase in calcein and DOX permeation. DOX treatments resulted in moderate epidermis accumulations, with epidermis/SC ratios of 2 for non-ionic hydrogels and SLC treatments, 1.4 for Chitosan, and 0.7 for Carbopol, respectively. In conclusion, the authors suggested that surface pre-seeding with CN could further improve the number and distribution of LTRs. Another investigation measuring the potential effects of low frequency (20 kHz) on skin permeability was evaluated by Aldwaikat and Alarjah et al. [85] in which a model system consisting of the anti-inflammatory drug diclofenac sodium (DS), the skin substitute EpiDerm™, and the Franz diffusion cell was employed. Upon examination, the system revealed that the applied parameters of 20 kHz (continuous mode) at 10% and 20% amplitudes for 5 min increased DS permeation to 263% and 546%, respectively, compared to the control. Amplitudes above 20% in continuous mode induced cellular damage (not with pulse mode) and complete shredding of the SC at 50%, suggesting further efforts to improve percutaneous transport via the augmentation (increase in duration and intensity) of SF may result in the frequency of adverse skin reactions [11].

Initial efforts with dual-frequency (DF) (20 kHz and 1 MHz) sonophoresis to minimize detrimental skin reactions and improve percutaneous transport have delivered some promising successes [86,87]. The early studies by Schoellhammer et al. showed that the AC generated from lower frequencies (20–40 kHz) produced an increase in the number of pitted areas [87], whilst the intermediate frequencies (1 MHz or 3 MHz) resulted in a greater pitted area, encouraging more efficient transport. Upon examination, DFS enhanced the transportation of glucose in vitro and in vivo when compared with single frequencies, with both approaches inducing a similar degree of epidermal disruption. More recently, DFS has emerged as a treatment modality for a variety of inflammatory skin disorders, including Rosea [88] and Acne Vulgaris [89]. Furthermore, the additional cavitational activity provided by DFS has been shown to stimulate progenitor cell differentiation [90] and enhance the transdermal delivery of minoxidil, the hair growth enhancement drug [91]. These advancements highlight the versatility of ultrasound technologies in dermatological applications.

### 3.1. Skin Infection

**Figure 3:**
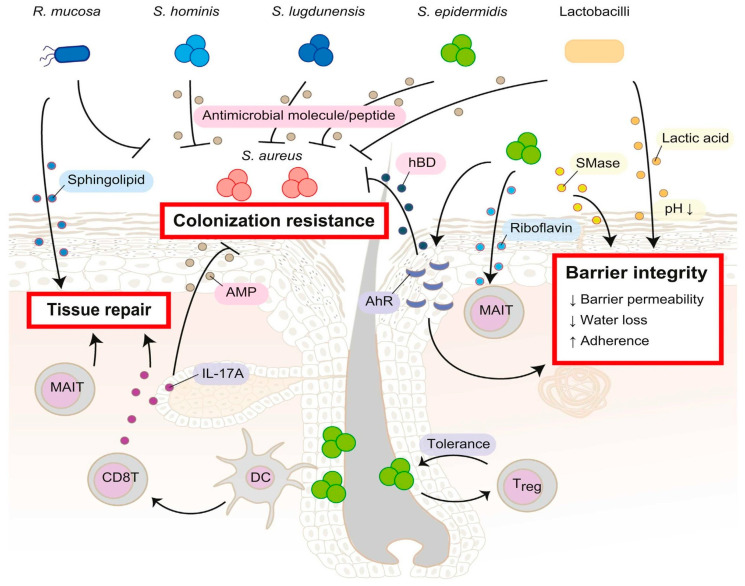
Regulating barrier function. Host–microbiota interactions involve complex mechanisms among species like *Staphylococcus hominis*, *S. lugdunensis*, *S. epidermidis*, *lactobacilli*, and *Roseomonas mucosa*, which secrete antimicrobial peptides (AMPs) inhibiting *Staphylococcus aureus*. *S. epidermidis* interacts with dendritic cells via hair follicles, stimulating IL-17A-producing CD8+ T cells to boost host AMP secretion and tissue repair. It also induces regulatory T cells (Tregs) early in life, fostering tolerance to commensals. The aryl hydrocarbon receptor activation by *S. epidermidis* drives keratinocyte production of β-defensin, preserving barrier integrity. Its sphingomyelinase supports ceramide synthesis in the stratum corneum. *R. mucosa* aids tissue repair by producing sphingolipids and suppressing *S. aureus*. Riboflavin from microbes activates invariant T (MAIT) cells for tissue repair, while lactobacilli secrete lactic acids, acidifying the skin and inhibiting pathogens. Reproduced with permission [94].

Fifty percent of patients with primary skin infections experience recurrences within six months, even when treated with appropriate antibiotics and infection control measures [92]. The skin microbiome is the first line of defense against infection, comprised of 19 phyla and 1000 microbial species. These species form regionally specific communities whose composition is dependent on the local environment and underlying skin physiology; for example, bacteria species (*Staphylococcus* and *Corynebacterium*) are prevalent in moist areas (folds), whereas lipophilic Propionibacterium species proliferate in regions (the head, neck, and chest) with the highest surface area (sebaceous) and follicle density [93,94,95,96]. Collectively, these bacteria ensure barrier integrity by participating in tissue repair, keratinocyte development, and colonization resistance (Figure 3). Loss of barrier integrity can occur via reduced *S. epidermidis* stimulation of the aryl hydrocarbon receptor followed by a downturn in keratinocyte human β-defensin 3 (HBD-3) production [97], rendering the skin susceptible to SA colonization and internalization. HBD-3 is an antimicrobial peptide and, along with cathelicidin (LL-37), restricts the growth of *S. aureus* and group A Streptococcus [97].

Community adjuvants in support of HBD-3 activity are soluble phenol modulins (PSMs) γ and δ provided by *S. epidermidis*, coagulase-negative staphylococci (CoNS). A potential fall in PSM levels resulting from SA colonization can be compensated via a combination of US and HBD-3 microbubbles. Work (in vivo and in vitro) by Li et al. and Zhu et al. [98,99] showed that when combined with US (SonoVue) microbubbles, HBD-3 can sufficiently promote icaR expression whilst inhibiting the biofilm genes icaAD and MecA. Similarly, Zhou et al. [100] also reported that HBD-3 US (SonoVue) microbubbles were twice as effective in reducing MecA expression than HBD-3 alone.

Previous theories regarding the synergistic mechanisms between microbubbles, AMPs, and antibiotics responsible for enhanced sensitivity, reduced genetic expression, and biofilm dispersion remain in their infancy, requiring additional investigations [101,102]. To address these gaps, Lattwein et al. employed a combination of real-time high-resolution fluorescence imaging and multiple acoustic parameters [102]. Their study aimed to elucidate these interactions by monitoring the direct effects of 2 MHz on SA biofilms with and without non-targeted microbubbles or the antibiotic (oxacillin 1 ug/mL) at the single-cell level. The acoustic parameters (high pressure and cycles) used in the study led to high rates of dispersion and biofilm removal (87%). Bacterial dispersion dominated sonoporation, with most of the former attributed to oscillating microbubbles and not antibiotic presence. However, the authors noted that sonoporation of these parameters would be difficult to track at the single-cell level and may have contributed to a decrease in the uptake of propidium iodide (PI) encountered for all treatments, with clustering remaining problematic, although a microbubble concentration was chosen to maximize sonoporation potential. In summary, the researchers suggested that to better understand microbubble SA relationships, real-time observational experiments of targeted microbubble (vancomycin or affirmer protein) [102] behaviors in microcirculatory flows [103] would be required.

The type of microflow found in vivo can be replicated in vitro via a microfluidic platform, as demonstrated by Caudwell et al. [103]. In their study, a triple-chambered platform with confocal fluorescence laser scanning microscopy (CLSM) was employed to monitor the growth of SA biofilms and evaluate the binding potential of nonspecific and specific affimer-decorated MBs.

Affimers are small heat-stable proteins (~12 kDa) serving as antibody replacements and are rendered “specific” with the inclusion of an active targeting sequence. In this case, the specific affimer was AClfA1, designed to target the biofilm adhesion protein ClfA1. Without the application of US, MBs decorated with AClfA1 showed an 8-fold increase in binding compared to MBs functionalized with the control affimer and were resistant to flow rates up to 250 μL/min. When subjected to 2.25 MHz US (pulse repetition rate of 10 kHz) for 2 s, the decorated biofilm lost 25% of its mass with an 8% increase in dead cell numbers. Interestingly, the polystyrene standardization beads decorated with AClfA1 showed a 9-fold increase in binding. This suggests solid nuclei such as Poly(lactic-co-glycolic acid) PGLA and gold nanoparticles could be effectively functionalized with affimers for similar applications [16].

Integrating the precision of microfluidics with labeled NBs represents a crucial stage in improving our understanding of the mechanisms of sonoporation. Moreover, microfluidic systems are highly compatible with micro-optics and piezoelectric architectures (linear, tapered, slanted, and focused transducers) and allow us to model and examine the flow of microbubbles in microvascular systems with varying O_2_ concentrations [104]. For those readers interested in exploring the benefits of pairing microfluidic devices with ultrasound, the recent review by Huang et al. is suggested [105]. These developments underscore the versatility of ultrasound technologies in addressing challenges across a range of biological contexts.

### 3.2. Role of the Ica Operon in Biofilm Recurrence

The ica operon, consisting of the genes icaADBC, encodes enzymes essential for the synthesis of polysaccharide intercellular adhesin (PIA), a critical component of the extracellular polymeric substance (EPS) matrix in *Staphylococcus aureus* biofilms. In chronic wounds, biofilm formation significantly hinders the healing process by protecting bacteria from antimicrobial agents and host immune responses [106,107]. The ica operon plays a crucial role in biofilm development, with PIA facilitating cell-to-cell adhesion and biofilm maturation.

In the context of chronic wounds, biofilm recurrence presents a major clinical challenge. Even when initial debridement or antimicrobial therapy successfully disrupts the biofilm, residual bacterial populations, particularly persister cells, can rapidly re-establish biofilms. The ica operon is central to this process, as PIA production enhances bacterial adhesion and enables the quick reconstruction of biofilm architecture in the wound bed [7].

The expression of the ica operon is tightly regulated by icaR, a transcriptional repressor that modulates icaADBC activity in response to environmental conditions. In chronic wound environments characterized by nutrient availability and immune system evasion, icaR repression is often lifted, promoting PIA production and facilitating biofilm reformation. Studies have demonstrated the efficacy of combining ultrasound therapy with antibiotics to mitigate biofilm recurrence. For instance, low-frequency ultrasound (50 kHz) in combination with antibiotics has shown significant reductions in *S. aureus* biofilms, achieving up to 93% reductions within two hours of treatment and up to 97% with extended treatment durations [108]. Another study explored the use of microbubble-mediated ultrasound to enhance antibiotic delivery, showing that cavitational activity facilitated the deeper penetration of daptomycin into biofilms, leading to faster bacterial clearance and improved outcomes in wound models [109].

Preventing biofilm recurrence in chronic wounds requires a multifaceted approach. Ultrasound therapy, for example, disrupts biofilm integrity through cavitational forces while enhancing antibiotic penetration. Combining this with agents that inhibit ica operon activity or prevent PIA synthesis could further mitigate the risk of biofilm re-establishment [108]. Additionally, adjunctive therapies targeting persister cells and quorum-sensing pathways, such as the accessory gene regulatory (agr) system, offer potential strategies to break the cycle of biofilm recurrence [110].

### 3.3. Intracellular Infection

One of the first cell types to succumb to SA internalization is keratinocytes. To achieve this, SA utilizes the actions of numerous pore toxins (α-hemolysin, Leukocidins ED, HlgCB, HlgAB, SF-PVL, AB, and MF); the most notable of these is the pore-forming toxin α-hemolysin, resulting in ion imbalance. In concert with these toxins, Protein A binds to tumor necrosis factor (TNF) surface receptors, inducing a sustained (T-helper)Th2 polarized response via Langerhans cells. The Th2 inflammatory response is further enhanced by the actions of SA proteases, which induce thymic stromal lymphopoietin in the keratinocytes, resulting in the internalization of SA.

In addition to keratinocytes (14–30 μm), other cell types susceptible to *S. aureus* internalization include fibroblasts (1–4 μm), osteoclasts (150–200 μm), and epithelial (8–21 μm) and endothelial (50–70 μm, across) cells. These cells act as a haven for SA bacteria, shielding them from oral antimicrobials and the immune system, permitting reproduction and genetic exchange in the cytoplasm. Such havens or reservoirs are thought to play a role in infection recurrence. Moreover, many of these cells are susceptible to intracellular infection from other species, such as *Salmonella*, *Klebsiella pneumonia,* and *Escherichia coli (E. coli)*, to name but a few [111,112,113]. Successful targeting of these intracellular pathogens depends on the intracellular concentration of the active drug and its retention [114]. A study by Horsley et al. showed that a combination of US and gas-filled microbubbles decorated with gentamicin-loaded liposomes can significantly enhance the delivery of drugs into the cytoplasm of apical cells. The ultrasonic bubbles delivered twice the concentrations than liposomes alone and over 16 times the concentrations achieved via free diffusion. A 20 s ultrasonic exposure (1.1 MHz, 2.5 Mpa, 5500 cycles, and 20 ms pulse duration) with this novel therapy was as potent as 2 h of 200 μg/mL free gentamicin, achieving a 75% reduction in bacteria burden compared to free gentamicin alone [115].

In addition to keratinocytes, SA can persist within pro and anti-inflammatory macrophage phenotypes M1 and M2. In the acute stages of infection, SA employs a series of countermeasures to the high ROS activity and heightened cytokine (IL-1β, IL-6, and IL-12) secretion associated with M1 phagocytosis, such as enhanced catalase and superoxide dismutase activity, cytokines IL-10, -4, and -13, and the activation of phosphoinositide 3-kinase (PI3K)/Akt [116]. However, M1 infection control could be compromised (impaired phagosome maturity) if the infective strain is highly virulent or the host suffers from an underlying skin disease (psoriasis, atopic dermatitis, or acne). In such cases, the immuno response may be weighted against the M2 phenotype, allowing the M1 subtypes to dominate [117]. In addition, SA can also modulate the nitric oxide levels of M2 subtypes by suppressing inducible nitric oxide synthase (iNOS), allowing the pathogen to hijack the phenotype’s metabolic machinery, thereby providing a suitable intracellular environment for replication. Taken together, SA can impair both phenotypes, prolonging infection and recovery.

At high doses, the application of low-intensity pulsed ultrasound (LIPUS) (1 MHz, 1 h exposure at 65 mW/cm^2^, 10% duty cycle, and pulses of 300 μs) can encourage keratinocyte inflammation, NF-κB activation, and IL6 elevation [118], prolonging the inflammatory phase. On the other hand, lower-dose LIPUS, when coupled with anti-inflammatory drugs [119] and sonosensitizers (Chlorin e6 (Ce6)) [120], can be used to restore the phenotypic balance by promoting M1–M2 transitions. Therefore, applying a high–low or, vice versa, LIPUS dosage regime could potentially reduce infection-related phenotypic imbalances observed in acute and chronic stages of SA infection.

## 4. Methods of Delivery

The microbubbles produced by ultrasonic cavitation (UC) can exhibit variable size distributions, positions, and distances from the cells, all of which directly affect the delivery efficiency of a drug. A successful attempt to compensate (real-time feedback, minimizing adverse effects) for these variables via a combination of reference and application probes was demonstrated by de Marr et al. using clinical US systems in order to confirm US parameters and accelerate the translation of US and microbubble (USMB) therapies [121]. However, in that case, and many others (previous sections), 1–3 cm diameter probes were used to ensure focused USMB delivery. Moving from a “focused” to a highly focused delivery might require a form of evaporation (e.g., printer nozzle) or a tapered flow (needle or microneedle). Given patients’ needle aversions (pain), a non-invasive microneedle might be a better choice.

### 4.1. Microneedles and Ultrasonics

Microneedles can be fabricated and patterned from a vast array of materials (metals, soft or hard polymers, ceramics, composites, and biomaterials), making them attractive to many fields of science, including biology, medicine (neurology, diagnostics, and disease dermatology), and materials and engineering [122,123,124,125,126,127,128]. In the lab, microneedles come in various types (conical, pyramidal, sharp, hollow, dissolvable, and coated) and lengths (100–1200 μm) based on their application, the skin’s target depth, and the type of drug or therapeutic agent being delivered.

Microneedles (MN) can simultaneously mitigate the systemic toxicity of vancomycin and reduce the risk of MSRA resistance by strategically delivering the drug in a minimally invasive manner to the skin. A recent example of this is the work by Abu Ershaid et al. [129], in which double-casted (DC) sodium alginate MN loaded with vancomycin demonstrated a delivery percentage of 35% through full-thickness neonatal porcine skin, with 10% remaining 24 h later. With a delivery enhancement of >a factor 4 compared to previous reports (8%) [130], the authors concluded that double-casted sodium alginate MNs loaded with vancomycin were a viable drug delivery platform for SSTIs. MNs can also penetrate and disrupt the integrity of biofilms, exposing more internal surface area to antimicrobial agents. However, biofilm disruption can release virulent planktonic cells and proinflammatory free nucleic acids (NA). Through a combination of chemo and photodynamic therapies, Li et al. demonstrated that a microneedle patch encapsulating antibacterial (Fe/PDA@GOx@HA) nanoparticles (NP) and NA-absorbent amine-modified mesoporous silica NPs at the tips and bases could eradicate bacterial infection and expedite wound healing by modulating macrophage polarization and the removal of NAs from the infected site [131]. Further reductions in nonspecific inflammation and improvements in targeting potential are achievable by coating microneedles with a cell/biomimetic membrane. Purified biomimetic coatings are well-established in drug delivery research, providing unique homologous targeting effects [132]. An example of their application in skin diseases was demonstrated by Jing et al. [133], in which the enhanced targeting function of water-soluble (Karaya gum) microneedles coated with (epidermal) HaCaT cell membrane-liposomes (shikonin) was used in the treatment of imiquimod-induced psoriatic epidermal hyperplasia.

The surface of microneedles, like microbubbles, can be coated with a variety of organic (hematoporphyrins, photoporphyrins, and phthalocyanine) and inorganic nanomaterials (TiO_2_ BaTiO_2_, TiN, VS_4_) that, under US, produce ROS species. Such materials are named sonosensitizers, and, depending on stability (charge lifetime) requirements (stage of infection, low O_2_), a material like TiO_2_ with low toxicity and catalytic activity may be chosen. Ouyang et al. [134] theorized that TiO_2_’s low catalytic activity was due to its phase composition. Upon investigation, researchers found the anatase-brookite TiO_2_ (AB) phase was optimal and, when delivered as a nanoparticle via an MN hyaluronic acid (HA) patch, could eradicate 99.94% of an *S. aureus* biofilm after 15 min of ultrasound. The authors attributed AB potency to its high catalytic turnover (ROS production) due to efficient electron transfer (homointerface) and separation of US-generated electron–hole pairs, distinguishing it from other types anatase-rutile (AR), anatase-brookite-rutile (ABR) of TiO_2_.

**Figure 4:**
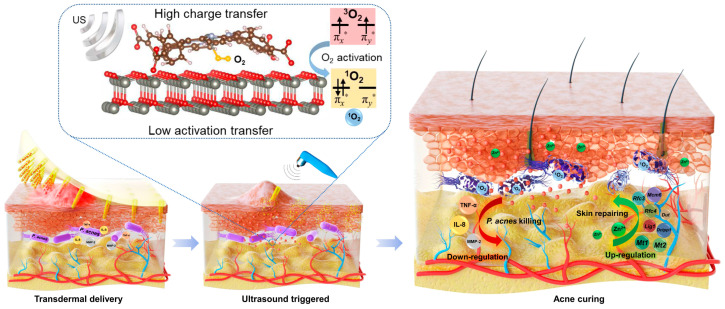
Sonocatalytic acne treatment via efficient ultrasound ion therapy with MN patch. Reproduced with permission [135] American Chemical Society.

Despite their versatility, metal–organic frameworks (MOFs) often suffer from inefficient electron transfer and separation, resulting in a weak response to ultrasound. A stronger response can be achieved by pairing the MOF with a semiconductor in a composite structure. By virtue of the material interface, the composite structure exhibits enhanced ROS production, lowering the US requirements (dosage). Of note is the multifunctional zinc porphyrin-MOF and zinc oxide (ZnTCPP@ZnO) nanoparticle composite, as proposed by Xiang et al. [135]. The researchers postulated that its exposure to US within an integrated microneedle patch would result in elevated ROS and the concomitant release of Zn2+ ions, triggering intra-cellular repair pathways within skin cells (Figure 4).

Upon investigation, the (10 × 10 mm) and (16 × 16 MNs) integrated patch demonstrated an in vitro oxygen-mediated antibacterial efficiency of 99.73% for *Propionibacterium acnes* (*P. acnes*) and 99.64% for MRSA after 15 min (1 W cm^–2^, 1 MHz, and 100% duty cycle) of US, respectively. Furthermore, the results suggested that ZnTCPP@ZnO was the main factor in driving gene differentiation and that the differential genes associated with replication and repair were correlated with Rfc4 and corresponding expression levels of Mt_1_ and Mt_2_ as initially postulated. However, if there is a critique, using a single probe in the animal study might be one. As suggested in the recent review by Peng et al. [136], the inclusion of internal reference needle/s could be an option going forward. Alternatively, the limitations of a separate probe could be mitigated with the incorporation of multiple transduction elements (piezoelectric)discs (PZT-Ds)) and corresponding fluidic chambers (cups) into a single device or a conformable patch [137]. Moreover, including thermal control would provide an additional reference, allowing researchers to distinguish permeation enhancements related to ultrasound and those related to heat. With such an experimental setup, Yu et al. reported decreases in skin resistance values of 62.3% for US (0.2–1.0) MHz and 22.7% for heat, respectively. Skin permeability studies using a porcine model and niacinamide resulted in 26.2- and 19.2-fold increases in drug transport with four-disc and single-disc patch configurations after 10 min of US compared to passive diffusion. Finally, the authors bravely noted that the power requirements for the 2D array patch (4 PZT-D elements) ranged from 3.6–4.8 W, while those for a single PZT-D element ranged from 1.05–1.3 W., highlighting the only deficiency, if any, in the device.

In addition to US, microneedles have shown universal compatibility with other technologies, such as iontophoresis, electroporation, and phototherapy, in the assisted transdermal delivery of drugs [138,139,140].

### 4.2. Piezoelectric (Nano-Enzyme): Biofilm and Wound Healing

Including its role in cell migration, ZnO functions as a third-generation semiconductor and a piezoelectric material [141,142]. Piezoelectric materials polarize under applied stress (acoustic or mechanical pressure), displacing positive and negative charges from the inside to the upper and lower surfaces of the material. In the case of ZnO, hexagonally symmetric wurtzite crystal structures provide the material with good piezoelectric and ferroelectric properties. The properties of ZnO can be further enhanced when combined with other materials (ceramic) or biomaterials, such as nanoenzymes. Nanoenzymes are materials with excellent stability that mimic the action of enzymes, some of whom have peroxidase activity and ROS potential. However, poor catalytic activity offsets their excellent stability, limiting their use in antibacterial applications. Ideally, a hybrid nano-enzyme with piezo and catalytic properties is required. With that in mind, Bail et al. [143] combined ZnO with Graphdiyne (GDY), realizing a nano-rod (NR)/sheet (ZnO@GDY NR) composite optimized for H_2_O_2_ decomposition. When irradiated with ultrasound (1 W cm–2, 1 MHz, and 100% duty cycle), the composite degraded rhodamine B (RhB) at a reaction rate constant 5 times that of ZnO NR, which increased to 14 in the presence of supplementary H_2_O_2_. Upon antibacterial testing, the composite of ZnO@ GDY NRs under US demonstrated decreases of 5.9 and 5.7 orders of magnitude in MRSA and *Pseudomonas Aeruginosa* colony numbers, equivalent to bacterial killing rates >99.999%, with similar rates reported in an in vivo mouse model as well.

For the most part, MOFs alone possess a highly symmetrical structure, which prevents their direct utilization in piezoelectric applications without substantive modification. An exception to this is the abundant asymmetric geometries found in UIO-66 that allow for easy modification and integration with organic ligands or metal ions (Zr to Hf), improving its conductivity. Moreover, when combined with a noble material (Ag, Au, or Cu) with an intrinsic preference for charge separation over recombination, the charge distribution of UIO-6 is markedly polarized, enhancing piezoelectric activity, as reported by Cai et al. [144]. Using a combination of UIO-66 and bioactive Au nanoparticles (nanoenzyme), the authors demonstrated a 2-fold increase in the intrinsic catalase and peroxidase activities of Au NPs, signifying their potential usage in low-O_2_ environments encountered in tumors and biofilms.

**Figure 5:**
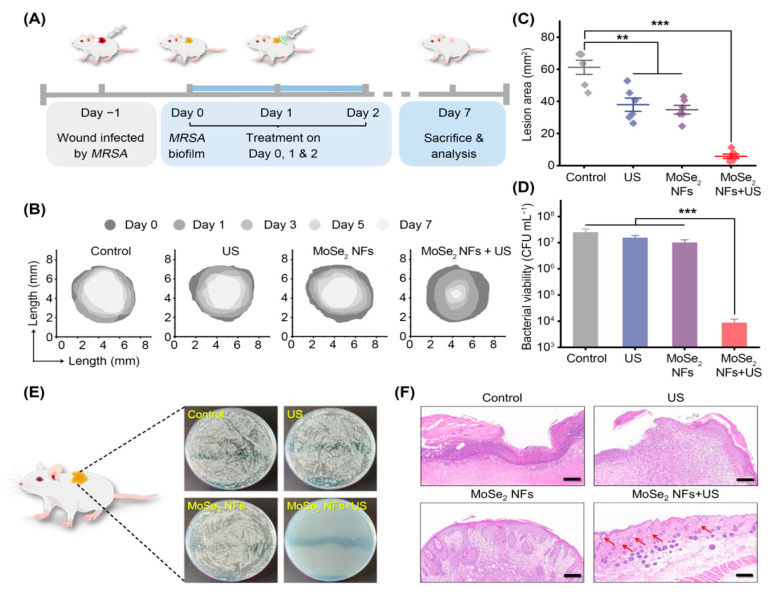
In vivo biofilm eradication and bacterial killing by MoSe_2_ NFs: (**A**) experimental schedule; (**B**) lesion traces over 7 days; (**C**) relative lesion area variation after 7 days; (**D**,**E**) MRSA viability at infection sites; (**F**) H&E staining of skin tissues (scale bars = 200 μm; arrows indicate hair follicles). Error bars represent SD (n = 6), with significance levels ** *p* < 0.001 and *** *p* < 0.0001. Reproduced with permission [145] from the American Chemical Society.

The regulatory and antioxidant roles of reduced glutathione (GSH) are key to a biofilm’s growth and maturation. Mirroring the cascade systems found in biology, researchers proposed a piezoelectric/enzymatic system for the dual-driven catalytic eradication of MDR bacterial biofilms using MoSe_2_ nanoflower (NFs) combined with low-intensity (0.3 W) US. Possessing a high curvature and large surface area, MoSe_2_ NFs were expected to exhibit outstanding piezoelectricity and GSH-biomimetic activity. Upon in vitro investigations, US-induced MoSe_2_ NFs demonstrated broad spectrum potential, yielding 3.8 log^10^, 4.0 log^10^, and 4.0 log^10^ bacterial reductions in MRSA, SA, and *E. coli* biofilms, respectively. The authors attributed the performance of MoSe_2_ NFs to three factors: first, the attraction of positively charged MoSe_2_ NFs to the negative biofilm and enhanced permeation under US irradiation; second, the oxidation of glutathione (GSH) via MoSe_2_ NFs to GSSG reduces the biofilm’s ability to neutralize ROS, limiting growth; and third, MoSe_2_ NFs’ dual-driven catalysis generated excessive OH•, leading to biofilm dissemination and bacterial killing [145]. In addition, the antibacterial potential of MoSe_2_ was further assessed over a seven-day period in vivo, using an abscess wound mouse model created from a local infection of MRSA (Figure 5).

On day 7, infected mice treated with US or MoSe_2_ NFs alone showed a reduction of 20% in lesion area, whereas a 90% reduction was observed in mice subject to combinatorial treatment. Moreover, the low US intensity coupled with the scavenger potential of the polyethyleneimine (PEI) coating at neutral pH minimized the off-target damage in favor of the wound healing process. Other examples of antibacterial sonodynamic therapies utilizing nanoenzymes and piezocatalysis in the treatment of secondary complications/infections arising from the migration of *S. aureus* and associated toxins from the skin to the deeper tissues can be found in the work by Sun et al. [146] and Xu et al. [147]. In addition, numerous and extensive reviews have recently addressed the interplay between biofilms and wound (chronic and acute) treatments [148,149,150,151,152,153].

## 5. Future Directions

**Figure 6:**
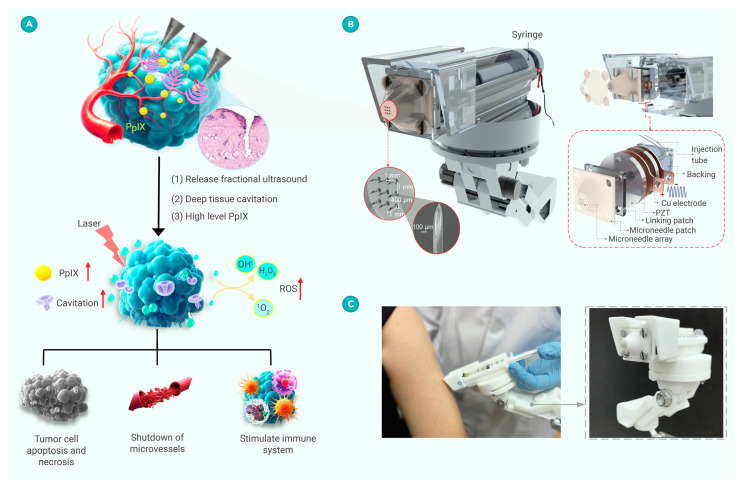
Design and mechanisms of PDMN-PDT for skin disease therapy. (**A**) Schematic representation of PDMN-PDT applied to lesions. (**B**) Diagram of PDMN structure (left) with an expanded view (right). (**C**) Image of the PDMN delivery system showcasing its flexibility in operation. Reproduced with permission [158] from Elsevier.

Currently, the industrial standard for portable biofilm eradication and wound repair is the MIST ultrasound spray system developed in 2006 [154] and is available through Sanuwave (https://sanuwave.com/ (accessed on 10 December 2024)). Since then, improvements have been made, such as the multi-nanoenzyme hydrogel spray combined with ultrasound proposed by Shang et al. [155], which has emerged as a promising alternative for treating diabetic wounds. In this case, the nanoenzyme hydrogel spray consisted of five enzyme-like activities—superoxide dismutase (SOD), catalase (CAT), glucose oxidase (GOx), peroxidase (POD), and nitric oxide synthase (NOS)—and when combined with US provided an all-in-one therapy accelerating in vivo diabetic wound healing. Accelerated wound repair has been taken a stage further by including Triboelectric nanogenerators (TENGs) in the field of self-power wound healing [156,157]. Moreover, a novel piezoelectric-driven microneedle platform (PDMN-operating frequency, 114 kHz, and 3 W.cm^2^) in the absence of sonosensitizers or nanoparticles (Figure 6) was recently evaluated in a small clinical trial (n = 25) for multiple skin diseases with varying thicknesses and architectures, suggesting a dawn in US skin therapies [158].

Finally, we note the antimicrobial treatment of *Staphylococcus aureus* using the Clustered Regularly Interspaced Short Palindromic Repeats (CRISPR)-Cas system. While significant progress has been made in modifying or silencing antibiotic resistance genes in model strains like USA300 using viral vectors [159,160], the integration of this approach with ultrasound (US) remains underexplored. Akin to the extensive development of CRISPR-based medical treatments in other fields, its potential synergy with ultrasound could represent a promising avenue for future antimicrobial research.

## 6. Conclusions

This review examined the potential of ultrasonic technologies as adjunctive therapies for managing *Staphylococcus aureus* skin infections. The plethora of available US frequencies and intensities has allowed researchers to employ a set of parameters geared toward the treatment of specific skin or wound infections. *S. aureus* is renowned for exhibiting resistance to various stressors, from the physical to the immunological, prolonging the infection and diseased state, which suggests that a combinatorial treatment approach should be employed. The coupling of US with antimicrobial-loaded CN (nano and microbubbles + antibiotics) has demonstrated potential in restoring host skin homeostasis and phenotypic balance, which reduces infection and recovery times. Moreover, this approach has shown potential in modulating the ica operon’s role in biofilm formation and targeting intracellular infections, reducing the risk of recurrence.

With the emergence of microneedle patches, piezoelectric devices and microbubble-enhanced ultrasound systems are finding traction in the field of chronic wound healing and biofilm recurrence. As non-invasive alternatives to sharp debridement, these refined delivery technologies have shown similar therapeutic efficacies, with some examples [135] demonstrating antibacterial and cellular repair properties, with others, such as the piezoelectric/enzymatic system, offering a near-complete solution to biofilm eradication and wound healing. The clinical success of these technologies relies on multidisciplinary collaborations to refine techniques, improve delivery mechanisms, and conduct robust studies to bridge the gap between the research lab and clinical applications.

As the global healthcare landscape increasingly grapples with antibiotic resistance, integrating ultrasonic technologies represents an innovative approach to the treatment of skin infections.
